# Colchicine for the Prevention of Major Adverse Cardiovascular Events After Acute Coronary Syndromes: A Systematic Review and Meta-Analysis of Large, Long-Term, Placebo-Controlled Randomized Trials

**DOI:** 10.3390/jcm15072695

**Published:** 2026-04-02

**Authors:** Roxana Mihaela Popescu, Ruxandra Dragoi Galrinho, Manan Pareek, Dharmaraj Karthikesan, George Dumitrescu, Șerban Mihai Balanescu, Dragoș Vinereanu

**Affiliations:** 1 University of Medicine and Pharmacy “Carol Davila”, Department of Cardiology, Elias Emergency University Hospital, 020021 Bucharest, Romania; george-mihai.dumitrescu0720@stud.umfcd.ro (G.D.);; 2 Department of Cardiology and Cardiovascular Surgery, University and Emergency Hospital, 05098 Bucharest, Romania; 3 Rigshospitalet—Copenhagen University Hospital, 2100 Copenhagen, Denmark; 4 Hospital Sultanah Bahiyah, Alor Setar 05460, Malaysia; 5 University of Medicine and Pharmacy “Carol Davila”, Department of Cardiology and Cardiovascular Surgery, University and Emergency Hospital, 05098 Bucharest, Romania; vinereanu@gmail.com

**Keywords:** colchicine, inflammation, acute myocardial infarction, major adverse cardiovascular events (MACEs), death, revascularization

## Abstract

**Background**: Despite major advancements in the treatment of post-acute coronary syndrome (ACS), the prevalence of early and late major adverse cardiovascular events (MACEs) remains high. Inflammation, a key feature of atherosclerosis, plays an important role in the healing process following ACS. This suggests that anti-inflammatory agents might improve both atherosclerotic progression and cardiovascular outcomes. Colchicine has potent anti-inflammatory effects and may, therefore, be a suitable agent for mitigating this response. **Methods**: We conducted a systematic search up to September 2025 across Embase, MEDLINE, the Cochrane databases, and the Clinical Trials.gov registry to assess whether colchicine administration after ACS reduces the risk of a MACE (a composite of cardiovascular death, ACS, stroke, and urgent revascularization). We selected placebo-controlled randomized trials enrolling more than 500 participants, in which colchicine was administered as a long-term intervention, defined as treatment and/or follow-up of at least 12 months, and in which MACEs were assessed as the primary endpoint. **Results**: We included three large, long-term, placebo-controlled randomized trials (n = 12,602 participants). Primary events occurred in 485 participants in the colchicine group and 551 in the control group, with a calculated odds ratio (OR) of 0.87 (95% CI 0.77–0.99, *p* = 0.03), with high heterogeneity between studies (I^2^ ≈ 71%): *p* for heterogeneity ≈ 0.03. Subgroup analysis of diabetic patients (OR 0.81, 95% CI 0.63–1.04), as well as of individual components of the primary outcome, showed non-significant effects: OR= 0.92 (95% CI 0.76–1.11, *p* = 0.38) for myocardial infarction, OR = 0.88 (95% CI 0.72–1.07, *p* = 0.15) for revascularization, OR = 1.09 (95% CI 0.86–1.38, *p* = 0.29) for cardiovascular death, and OR = 0.89 (95% CI 0.63–1.27, *p* = 0.47) for stroke. **Conclusions**: In this meta-analysis of large, long-term, placebo-controlled randomized trials, colchicine administration after ACS was associated with a modest reduction in MACEs. However, the proximity of the confidence interval to unity reflects a statistical equilibrium between opposing trial-level effects rather than a robust treatment signal. Further investigation is warranted, given the small number of existing large trials and their heterogeneity.

## 1. Introduction

Despite the remarkable recent improvement in prevention, diagnostic, and management strategies, acute coronary syndrome (ACS) remains a major cause of mortality and morbidity worldwide [1,2,3]. The spectrum of ACS ranges from unstable angina to ST elevation myocardial infarction (STEMI) and non-ST elevation myocardial infarction (NSTEMI). In most presentations of ACS, atherosclerotic plaque disruption or erosion is the predominant underlying mechanism, inflammation being one of the key features; however, non-atherosclerotic processes, including endothelial dysfunction and coronary vasospasm, may also contribute in specific entities, such as vasospastic angina and some MINOCA subtypes. Moreover, inflammation occurs both as part of the natural healing process and following reperfusion [4]. Inflammation contributes substantially to adverse outcomes in ACS, particularly in acute myocardial infarction (AMI), by promoting ischemia/reperfusion injury [5,6] and ventricular remodeling, which may ultimately lead to heart failure (HF). Therefore, the overall burden of inflammatory processes has important prognostic value and contributes to an increased risk of major adverse cardiovascular events (MACE) after ACS.

Colchicine, a well-established anti-inflammatory medication, targets the NOD-like receptor protein 3 (NLRP3) inflammasome [7]. Beyond its impact on myocardial inflammation, colchicine may play a role in inhibiting neointimal proliferation following local vascular inflammation and endothelial damage related to balloon or stent angioplasty [8]. After percutaneous coronary intervention (PCI), the anti-inflammatory properties of colchicine are also associated with reduced release of cytokines and other inflammatory mediators, thereby attenuating ischemia/reperfusion injury [8,9,10]. Apart from reducing plaque instability after AMI, colchicine inhibits platelet activation and improves endothelial function [9,10]. Nevertheless, the optimal timing, dosage, treatment duration, and estimation of treatment effect of colchicine administration following ACS remain subjects of ongoing discussion. Previous meta-analyses included RCTs with varied sample sizes and patients with diverse vascular pathology (ACS, stable coronary artery disease or stroke) [11,12].

Therefore, we performed a systematic review and meta-analysis focused on ACS, restricted to published randomized controlled trials (RCTs) with more than 500 participants. The aim of this study was to establish the efficacy and safety of colchicine in patients with ACS.

## 2. Materials and Methods

### 2.1. Protocol and Registration

This meta-analysis was conducted in accordance with the Preferred Reporting Items for Systematic Reviews and Meta-Analyses (PRISMA) guidelines [13]. The protocol was registered at PROSPERO (ID: CRD420251175300), 2025, https://www.crd.york.ac.uk/PROSPERO/view/CRD420251175300 (accessed on 15 September 2025).

### 2.2. Eligibility Criteria

We included placebo-controlled randomized trials with more than 500 participants that examined the effect of long-term colchicine treatment in adult patients (at least 18 years of age) with ACS, where long-term was defined as an intended treatment strategy and/or clinical follow-up of at least 12 months. Trials were eligible if MACEs constituted the primary outcomes. When more than one publication was available for the same trial, we extracted outcome data from the longest available published follow-up used for the primary endpoint. A typical MACE was a composite of myocardial infarction, stroke, cardiovascular death, and revascularization, while secondary outcomes were myocardial infarction, stroke, cardiovascular death, and revascularization assessed separately. No language or date restrictions were applied.

### 2.3. Information Sources

To investigate whether colchicine might be a valuable long-term strategy post-ACS, we conducted a systematic search across EMBASE, MEDLINE, the Cochrane Library, and ClinicalTrials.gov, from inception until October 2025. RDG and GD extracted the data independently and assessed the quality, while MRP resolved the discrepancies. We did not include conference abstracts, theses, or backward/forward citation chasing.

### 2.4. Search Strategy

The systematic search summary is available in Table A1. Database-specific search strategies are provided in the Appendix A (Table A2).

### 2.5. Study Selection

Two independent reviewers screened titles/abstracts and full texts (RDG, MRP); conflicts were resolved by a third reviewer (MP). Eligibility decisions were recorded according to the PRISMA guidelines (PRISMA checklist in Table A3) [13].

### 2.6. Risk of Bias Assessment

For the risk of bias assessment, the Cochrane Risk of Bias tool, version 2 (RoB 2), was used. Five domains were evaluated for each trial: randomization, deviations, missing data, outcome measurement, selection of reported results. Two independent reviewers assessed each domain; disagreements were resolved by consensus. Overall risk of bias (RoB) was assigned as the highest risk across domains.

### 2.7. Effect Measures and Synthesis

A tabular meta-analysis was performed using the Peto odds ratio (OR) method for weighted averaging [14]. Odds ratios were calculated with 95% confidence intervals, and forest plots were produced for MACEs and all secondary outcomes as components of the primary outcome. A *p*-value < 0.05 was considered significant. We performed a common-effect model as the main analysis and a random-effects model as sensitivity analysis, with heterogeneity assessed using I^2^. A Mantel–Haenszel common-effect model was used as a second method to evaluate the robustness of the primary analysis. For each eligible trial, data were extracted from the longest available published follow-up corresponding to the primary endpoint; therefore, the COPS trial contributed the 24-month follow-up data to the primary synthesis. To address variation in follow-up duration across the included trials, we performed an exploratory analysis stratified by the longest available follow-up used for the primary outcome synthesis. Using this approach, the eligible trials were grouped into two follow-up categories: approximately 24 months for COPS (prespecified 24-month follow-up) and COLCOT (median 22.6 months) and 36 months for CLEAR SYNERGY (OASIS-9). Because only two studies contributed to the ~24-month category and one study contributed to the 36-month category, these analyses were considered exploratory.

### 2.8. Small-Study Effects/Publication Bias

To evaluate publication bias, Funnel plots of log hazards ratio (HR) vs. precision were planned. Egger’s regression test was considered to assess small-study effects. A subgroup analysis of the diabetic population was performed, as well as a separate analysis of individual components of the primary outcome (MI, stroke, CV death, and revascularization). We sought to exclude trials for high risk of bias and performed a leave-one-out sensitivity analysis.

### 2.9. Certainty of Evidence

For the GRADE evaluation, RCTs started as “high” certainty and were downgraded for risk of bias, imprecision, inconsistency, indirectness, or publication bias. The Summary of Findings tables report absolute/relative effects, participant numbers, and GRADE certainty.

### 2.10. Software

The meta-analysis was performed using RStudio version 4.3.2.

## 3. Results

### 3.1. Study Selection

**Figure 1:**
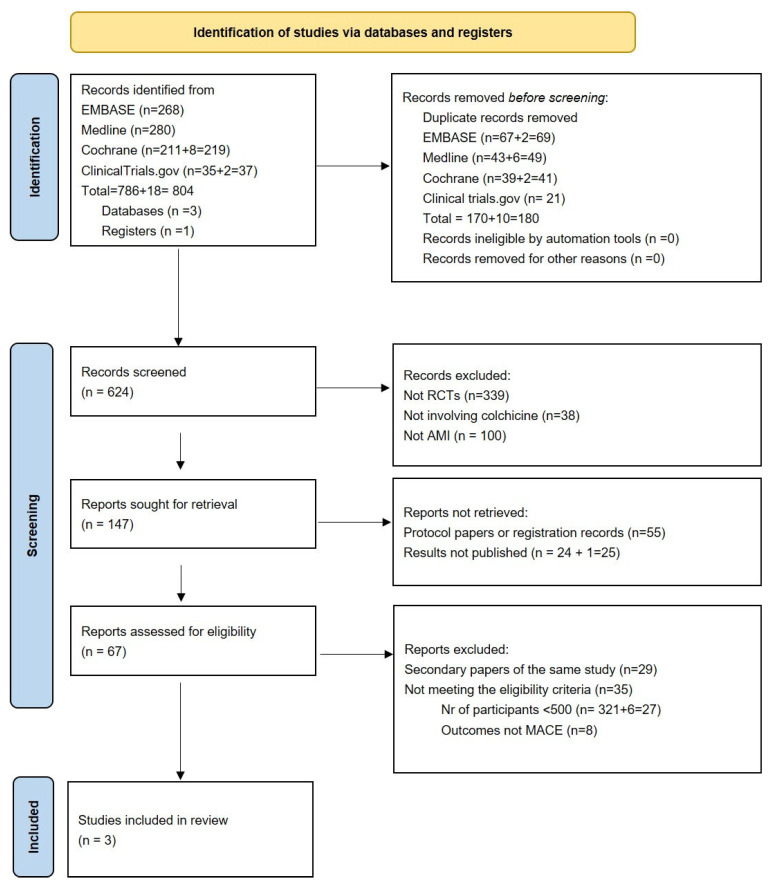
PRISMA flow diagram of systematic search results and study selection.

A total of three placebo-controlled randomized clinical trials met the inclusion criteria (see Figure 1) and were included in the analysis: Colchicine in Patients With Acute Coronary Syndrome (COPS), the Colchicine Cardiovascular Outcomes Trial (COLCOT), and Colchicine in Acute Myocardial Infarction (CLEAR SYNERGY (OASIS-9)) [15,16,17,18].

The systematic search identified several trials that did not meet all eligibility criteria, mainly due to a small sample size. However, they remain noteworthy due to the observed effect of colchicine on ACS patients and are summarized in Table A4.

### 3.2. Characteristics of Included Studies

The data from the selected trials were extracted, and the key trial features are summarized in Table 1.

### 3.3. Risk of Bias in Included Studies

Outcome-level RoB using RoB 2 domains was performed for all three trials. COLCOT showed low risk across all domains; centralized randomization; double-blinded, minimal missing data; and objective adjudicated endpoints. The CLEAR SYNERGY trial also showed low risk across all domains. It was a large, multicenter RCT, double-blinded, with objective clinical outcomes and negligible loss to follow-up. However, the COPS trial raised some concerns due to limited reporting of the two-year follow-up and mortality imbalance. The randomization and blinding were adequate, and the outcomes were objective, but the risk of selective reporting exists for long-term data (Table A5 and Table A6).

### 3.4. Certainty of Evidence (GRADE)

Overall certainty judgments for key outcomes are summarized in Table 2, while specific trial certainty of evidence is provided as Appendix C (Table A7, Table A8 and Table A9). The certainty of evidence was moderate for MACEs, myocardial infarction, and cardiovascular death and low for ischemia-driven revascularization and stroke. We downgraded for inconsistency where between-study heterogeneity was substantial (notably for revascularization and stroke) and for imprecision where confidence intervals spanned clinically important benefit to possible harm (e.g., stroke and revascularization).

### 3.5. Small-Study Effects/Publication Bias

As the inclusion criteria for the meta-analysis were studies with more than 500 participants, the small study effect was mitigated by design. Performing a funnel plot analysis for the detection of publication bias on only three studies was considered redundant.

### 3.6. Integrated Summary Statement

Across COLCOT, CLEAR SYNERGY, and COPS, the evidence was mixed (see Table 3). RoB2 was low in the COLCOT and CLEAR SYNERGY trials, with some concerns in COPS. Certainty ranged from low to high. The publication bias analysis showed no clear bias, but the small number of trials limited confidence. Caution was warranted in ACS populations, particularly regarding long-term safety. We did not identify serious concerns with risk of bias, indirectness, or publication bias across outcomes.

### 3.7. Primary Outcome Synthesis

**Figure 2:**
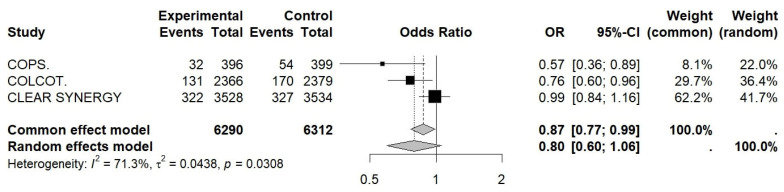
Forest plot of OR for the primary outcome (MACE). COPS, Colchicine in Patients With Acute Coronary Syndrome; COLCOT, Colchicine Cardiovascular Outcomes Trial; CLEAR SYNERGY (OASIS-9), Colchicine in Acute Myocardial Infarction (OASIS-9 trial).

The pooled effect of colchicine on MACEs was modest but statistically significant (OR= 0.87; 95% CI 0.77–0.99, z-score = 2.2, *p* = 0.036), with substantial heterogeneity between studies (I^2^ ≈ 71%; *p* for heterogeneity ≈ 0.03) (see Figure 2, Table A10). The number needed to treat (NNT) was approximately 114, meaning that about 114 patients would need to be treated over roughly 2 years to prevent one event. The verification method for the pooled odds ratio with a Mantel–Haenszel common-effect model yielded similar results (OR= 0.87; 95% CI 0.77–0.99): *p* = 0.037.

### 3.8. Sensitivity and Subgroup Analyses

#### 3.8.1. Secondary Outcomes

**Figure 3:**
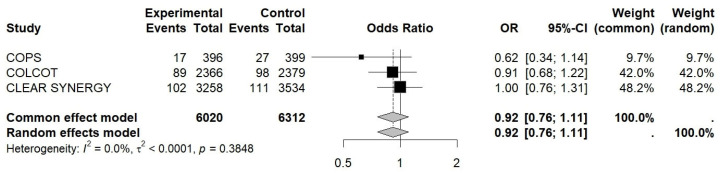
Forest plot of OR for new MI. Colchicine in Patients With Acute Coronary Syndrome; COLCOT, Colchicine Cardiovascular Outcomes Trial; CLEAR SYNERGY (OASIS-9), Colchicine in Acute Myocardial Infarction (OASIS-9 trial).

**Figure 4:**
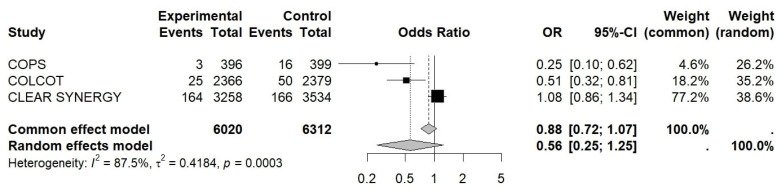
Forest plot of OR for revascularization. Colchicine in Patients With Acute Coronary Syndrome; COLCOT, Colchicine Cardiovascular Outcomes Trial; CLEAR SYNERGY (OASIS-9), Colchicine in Acute Myocardial Infarction (OASIS-9 trial).

**Figure 5:**
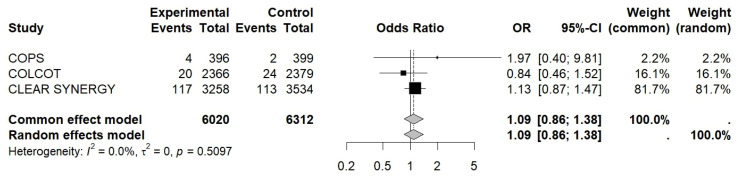
Forest plot of OR for CV death. Colchicine in Patients With Acute Coronary Syndrome; COLCOT, Colchicine Cardiovascular Outcomes Trial; CLEAR SYNERGY (OASIS-9), Colchicine in Acute Myocardial Infarction (OASIS-9 trial).

**Figure 6:**
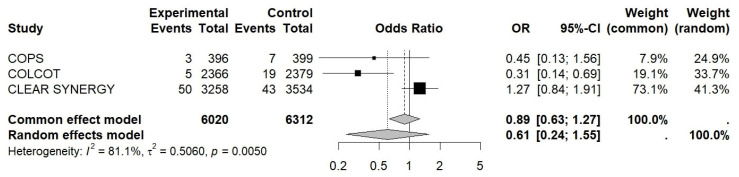
Forest plot of OR for stroke. Colchicine in Patients With Acute Coronary Syndrome; COLCOT, Colchicine Cardiovascular Outcomes Trial; CLEAR SYNERGY (OASIS-9), Colchicine in Acute Myocardial Infarction (OASIS-9 trial).

The OR for MI was 0.92 (95% CI 0.76–1.11, I^2^ ≈ 0.0%, *p* = 0.38), 0.88 for revascularization (95% CI 0.72–1.07, I^2^ ≈ 88%, *p* = 0.15), and 1.09 for CV death (95% CI 0.86–1.38, I^2^ ≈ 0.0%, *p* = 0.47), while for stroke, it was 0.89 (95% CI 0.63–1.27, I^2^ ≈ 81%, *p* = 0.29) (see Figure 3, Figure 4, Figure 5 and Figure 6).

Regarding the robustness of the model, the results were consistent between random-effects and common-effect models for MI and CV death (both I^2^ ≈ 0%), while differences were more pronounced for MACEs, revascularization, and stroke, in line with greater between-study heterogeneity.

#### 3.8.2. Subgroup Analyses

##### Diabetes Subgroup Analysis

**Figure 7:**
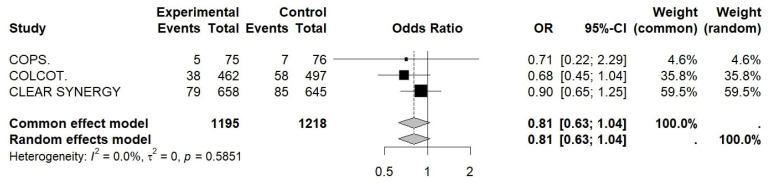
Forest plot of OR for pooled effects (MACE) in the diabetes subgroup. Colchicine in Patients With Acute Coronary Syndrome; COLCOT, Colchicine Cardiovascular Outcomes Trial; CLEAR SYNERGY (OASIS-9), Colchicine in Acute Myocardial Infarction (OASIS-9 trial).

In patients with type 2 diabetes, trial-level effects are provided in Figure 7. When pooled across studies, colchicine was associated with a non-significant reduction in MACEs (pooled OR 0.81, 95% CI 0.63–1.04, *p* = 0.09), with no between-study heterogeneity (I^2^ = 0.0%) (see Table A11, Figure 7).

##### Exploratory Subgroup Analysis According to Longest Available Follow-Up Duration

Supplementary analyses stratified by follow-up duration are presented in Table A13 and should be interpreted cautiously given the limited number of trials and non-uniform reporting of landmark time points. At approximately 24 months, both COPS and COLCOT favored colchicine. The prespecified two-year COPS follow-up reported 32 primary composite events among 396 colchicine-treated patients and 54 among 399 placebo-treated patients, corresponding to a significant reduction in the primary endpoint. COLCOT also showed a favorable effect estimate at a median follow-up of 22.6 months. Using the same event-count framework as the primary manuscript synthesis, the exploratory pooled estimate for the ~24-month stratum indicates a consistent signal of benefit at this time horizon (OR 0.71). At 36 months, CLEAR SYNERGY showed a neutral effect. These findings suggest that the apparent benefit was more evident at approximately two years than at three years, although the analysis remains descriptive and hypothesis-generating.

#### 3.8.3. Sensitivity Analyses

None of the studies were excluded for high risk of bias. The leave-one-out sensitivity analysis showed that removing each trial in turn did not materially change the pooled estimates. Excluding CLEAR SYNERGY generally shifted pooled effects toward greater benefit, whereas excluding COPS or COLCOT attenuated the apparent benefit. However, 95% CIs still overlapped with the primary analysis, suggesting that findings were robust to single-study exclusion (see Table A12).

## 4. Discussion

This meta-analysis was restricted to large, placebo-controlled randomized trials in order to mitigate, a priori, the small study effect and provide a focused estimate of colchicine use after acute coronary syndromes. Overall, colchicine administration after ACS was associated with a modest but statistically significant reduction in MACEs, with substantial heterogeneity across studies, suggesting that any potential benefit is modest and highly context-dependent. Nevertheless, with only three large, long-term, placebo-controlled randomized trials available, the results should be interpreted cautiously. Unlike broader meta-analyses that included smaller trials [19,20] and mixed vascular populations [11,12], the present analysis was deliberately restricted to large trials conducted specifically on ACS populations, thereby providing a more clinically focused estimate for this setting.

The pooled result reflects the balance between two favorable trials and one neutral trial. Overall, the result of the meta-analysis was driven by COLCOT and CLEAR SYNERGY trials, with a much larger sample size and number of events. CLEAR SYNERGY, as the largest and most contemporary trial, substantially attenuates the benefit observed in COLCOT. This suggests that earlier positive findings in ACS may not be applicable to a predominantly STEMI, PCI-managed population. Moreover, the CLEAR SYNERGY trial [18] showed a neutral effect of colchicine on MACEs after 3 years of administration. However, the follow-up period occurred during the COVID-19 pandemic, which might have negatively affected the collection of events through potential underreporting of non-fatal cardiovascular events. Moreover, subgroup analyses showed a trend toward benefit in the pre-pandemic cohort, which faded during and after the pandemic.

It is worth mentioning that dosing and timing varied between the analyzed trials. In COLCOT [17], a constant dose of 0.5 mg was administered starting within the first 30 days post-MI. In the COPS trial [15,16], a higher dose of 1 mg was administered during the index admission, which was then reduced to 0.5 mg and discontinued after one year. In the CLEAR SYNERGY trial, 0.5 mg of colchicine was started within the first 72 h post-MI [18]. Consequently, it remains uncertain whether early initiation of colchicine after ACS is necessary for MACE reduction and whether the low-dose regimen is sufficiently effective.

Although the diabetes subgroup analysis suggested potential benefit in COLCOT, these findings should be interpreted cautiously, as they are underpowered for definitive inference [21]. In the present meta-analysis, although the diabetes subgroup analysis did not reach statistical significance, the direction and magnitude of effect suggest a possible signal of benefit. This may reflect limited statistical power, and larger adequately powered studies are needed to determine whether patients with diabetes derive greater benefit from colchicine after ACS.

Across leave-one-out analyses, the direction of effect for colchicine on the primary outcome remained stable, with no single study reversing statistical significance in either the common-effect or random-effects model. Heterogeneity appeared largely driven by between-study differences anchored by the highest-weight trial, CLEAR SYNERGY, which showed an effect close to null compared with the more favorable estimates in COPS and COLCOT; removing CLEAR SYNERGY produced the largest reduction in I^2^. Overall, these checks support directional robustness but indicate that between-study variability is non-trivial and concentrated in one large study. GRADE certainty was moderate for the key outcomes of MACE, MI, and CV death, indicating that further research may influence but is unlikely to overturn these estimates. Certainty was low for revascularization and stroke, primarily due to substantial heterogeneity across trials and imprecise effect estimates, with wide confidence intervals. Taken together, these findings show uncertain clinical benefits of colchicine post-ACS, with the greatest residual uncertainty in revascularization and stroke.

The common-effect meta-analysis yielded a modest relative reduction (of 13%) in MACEs with colchicine versus a control. Interpretation of the common-effect estimate must consider that statistical significance does not equate to clinical certainty, particularly when heterogeneity is substantial. Clinically, the absolute benefit will be scaled with baseline risk (i.e., greater absolute reductions in higher-risk cohorts), but the confidence interval sits close to unity, underscoring a fragile margin of statistical significance.

These findings suggest that, while the overall direction favors colchicine, certainty hinges on study mix and patient context; replication in additional large, well-phenotyped cohorts with prespecified subgroup analyses would help clarify the subgroups where the benefit is the most consistent.

Current guideline positioning is consistent with this uncertainty. For chronic coronary syndromes, the recommendation in the 2024 European Society of Cardiology Guideline is clear. The addition of low-dose colchicine (0.5 mg daily) should be considered to reduce myocardial infarction, stroke, and need for revascularization for those with chronic coronary syndromes (Class 2a, Level of Evidence A) [22].

However, for ACS, according to the 2023 European Society of Cardiology Guideline, low-dose colchicine may be considered, particularly if other risk factors are insufficiently controlled or if recurrent cardiovascular disease events occur under optimal therapy (Class 2b, Level of Evidence A) [23]. Even if the CLEAR SYNERGY trial, published one year after the European guideline, failed to demonstrate a reduction in MACEs, according to the 2025 American College of Cardiology Guideline, low-dose colchicine may still be reasonable to reduce the risk of MACEs in patients after ACS (Class 2b) [24]. However, a specific timing is lacking, reflecting ongoing uncertainty in the literature.

While colchicine remains attractive due to its low cost and biological rationale, current evidence suggests that any benefit is likely modest and may be confined to specific subgroups or treatment strategies. Therefore, further research is needed to determine the role of colchicine in patients with ACS.

## 5. Conclusions

In this meta-analysis of large randomized placebo-controlled trials, colchicine administration after ACS was associated with a modest, statistically significant reduction in MACEs, with substantial heterogeneity across studies. The divergent results are likely due to differences in timing and dosing of colchicine, population, background therapies, adherence, and contextual factors, rather than a fundamental lack of efficacy. Moreover, selected populations or treatment strategies may derive more benefit; therefore, further trials focusing on patient selection, timing, and duration of therapy are required.

## Figures and Tables

**Table 1:** Characteristics of included clinical trials.

Trial	Design	N (I/C)	Population	Intervention (Dose)	Comparator	Follow-Up (Weeks)	Outcomes Measured
**COPS** **[15,16]**	Double-blind randomized placebo-controlled trial	795 (total)	Post-ACS managed with either PCI or medical therapy	Colchicine 0.5 mg twice daily for 1 month, then 0.5 mg once daily for 11 months	Placebo	12 months (~52 weeks) of treatment; prespecified 24-month follow-up	Primary: Composite of all-cause death, ACS (STEMI/NSTEMI/unstable angina), ischemia-driven urgent revascularization, and non-cardioembolic ischemic stroke
**COLCOT** **[17]**	double-blind trial	4745 (total)	Post-ACS	Colchicine 0.5 mg once daily (started within 30 days post-MI)	Placebo	Median 22.6 months (~98 weeks)	Primary: Composite of CV death, resuscitated cardiac arrest, MI, stroke, or urgent hospitalization for angina leading to revascularization
**CLEAR SYNERGY (OASIS-9)** **[18]**	Randomized placebo-controlled 2-by-2 factorial design	7062 (total)	STEMI or high-risk NSTEMI managed with PCI	Colchicine 0.5 mg once daily (started within 72 h after PCI for MI)	Placebo	Median 36 months (~158 weeks)	Primary: Composite of CV death, recurrent myocardial infarction, stroke, or unplanned ischemia-driven coronary revascularization

Abbreviations: COPS, Colchicine in Patients With Acute Coronary Syndrome; COLCOT, Colchicine Cardiovascular Outcomes Trial; CLEAR SYNERGY (OASIS-9), Colchicine in Acute Myocardial Infarction (OASIS-9 trial).

**Table 2:** Summary of findings (GRADE) population: adults with recent acute coronary syndrome (post-ACS). Intervention: low-dose colchicine. Comparator: placebo + standard of care. Setting: placebo-controlled randomized controlled trials (COPS, Colchicine in Patients With Acute Coronary Syndrome; COLCOT, Colchicine Cardiovascular Outcomes Trial; CLEAR SYNERGY (OASIS-9), Colchicine in Acute Myocardial Infarction (OASIS-9 trial).

Outcome	Participants (Studies)	Follow-Up	Common Effect	Certainty (GRADE)	Plain-Language Summary
**Major adverse cardiovascular events (MACEs)**	12,602 [3]	22.6–36 months (range across included trials)	OR = 0.87 [0.77; 0.99]	Moderate ⊕⊕⊕⊝ (downgraded **once** for inconsistency: substantial heterogeneity, I^2^ ≈ 71%, with differing effects across trials).	Little to no difference; confidence interval includes no effect.
**Myocardial** **infarction**	12,602 [3]	22.6–36 months	OR = 0.88 [0.73; 1.07]	Moderate ⊕⊕⊕⊝ (downgraded **once** for imprecision: confidence interval includes important benefit and no effect).	Little to no difference; confidence interval includes no effect.
**Ischemia-driven revascularization**	12,602 [3]	22.6–36 months	OR = 0.84 [0.69; 1.03]	Low ⊕⊕⊝⊝ (downgraded **twice** for inconsistency: very high heterogeneity, I^2^ ≈ 83%, and for imprecision: very wide confidence interval including substantial benefit and possible harm).	Little to no difference; confidence interval includes no effect.
**Cardiovascular death**	12,602 [3]	22.6–36 months	OR = 0.84 [0.69; 1.03]	Moderate ⊕⊕⊕⊝ (downgraded **once** for imprecision: confidence interval includes both small benefit and small harm).	Little to no difference; confidence interval includes no effect.
**Stroke**	12,602 [3]	22.6–36 months	OR = 0.88 [0.61; 1.27]	Low ⊕⊕⊝⊝ (downgraded **twice** for inconsistency: substantial heterogeneity, I^2^ ≈ 77%, and for imprecision: few events with wide confidence interval).	Little to no difference; confidence interval includes no effect.

**Table 3:** Summary of efficacy, side effects, and certainty across trials.

Trial	Primary Outcome	Side-Effects	Certainty
**COPS** **[15,16]**	No significant benefit at 12 months, Significant reduction in the primary composite outcome at 24 months	Numerical mortality imbalance, not statistically significant at 24 months	Low-to-moderate certainty
**COLCOT** **[17]**	Significant MACE reduction	Increased GI adverse events	High-certainty
**CLEAR SYNERGY** **[18]**	Neutral MACE effect	Increased diarrhea	High-certainty

Abbreviations: COPS, Colchicine in Patients With Acute Coronary Syndrome; COLCOT, Colchicine Cardiovascular Outcomes Trial; CLEAR SYNERGY (OASIS-9), Colchicine in Acute Myocardial Infarction (OASIS-9 trial).

## Data Availability

The data supporting the findings of this study (extraction sheets and analysis code) are available upon reasonable request from the authors.

## Abbreviations

The following abbreviations are used in this manuscript:

ACS	Acute Coronary Syndrome
AMI	Acute Myocardial Infarction
CENTRAL	Cochrane Central Register of Controlled Trials
CI	Confidence Interval
CLEAR SYNERGY (OASIS-9)	Colchicine in Acute Myocardial Infarction (OASIS-9 trial)
COLCOT	Colchicine Cardiovascular Outcomes Trial
COPS	Colchicine in Patients With Acute Coronary Syndrome
COVID-19	Coronavirus Disease 2019
CRediT	Contributor Roles Taxonomy
CV	Cardiovascular
EMBASE	Excerpta Medica Database
ESC	European Society of Cardiology
GRADE	Grading of Recommendations Assessment, Development and Evaluation
HF	Heart Failure
HR	Hazard Ratio
I^2^	I-Squared (Measure of Heterogeneity)
MACEs	Major Adverse Cardiovascular Events
MEDLINE	Medical Literature Analysis and Retrieval System Online
MI	Myocardial Infarction
NLRP3	NOD-Like Receptor Protein 3
NNT	Number Needed to Treat
NSTEMI	Non-ST-Elevation Myocardial Infarction
OASIS-9	Organization to Assess Strategies in Ischemic Syndromes-9
OR	Odds Ratio
PCI	Percutaneous Coronary Intervention
PRISMA	Preferred Reporting Items for Systematic Reviews and Meta-Analyses
PROSPERO	International Prospective Register of Systematic Reviews
RCT	Randomized Controlled Trial
RoB 2	Risk of Bias Tool Version 2
SE	Standard Error
STEMI	ST-Elevation Myocardial Infarction
WHO	World Health Organization

## Appendix A

**Table A1:** Systematic search summary; systematic review and meta-analysis methodology.

Objective	To Investigate if Current Clinical Trials Answer the Question of Whether Early Colchicine Administration Reduces MACE After ACS.
**Data sources**	Systematic search in Medline, EMBASE, Cochrane Library, and Clinical Trials.gov registry of clinical trials from 1946 until October 2025.
**Trial eligibility**	Study design: Randomized clinical trials More than 500 participants Patient population: Patients with ACS (either STEMI or NSTEMI) Intervention: Long-term administration of colchicine Comparator: Placebo or standard medical therapy without colchicine Primary outcome: MACEs, defined as a composite of cardiovascular death, recurrent MI, stroke, or urgent coronary revascularization.
**Exclusion criteria**	Trial design other than RCTs
**Results**	Studies looking at the effects of low-dose colchicine after ACS on MACEs.
**Method of analysis**	Tabular meta-analysis using the Peto method for weighted average.
**Result of meta-analysis reporting**	Common effect/random effects models to estimate the OR with a 95% CI, with study heterogeneity.

**Table A2:** Search terms and database-specific search strategies (MEDLINE/Embase/Cochrane/CT.gov).

Embase Search	
1.	exp randomized controlled trial/
2.	Controlled clinical trial/
3.	random.ti,ab.
4.	randomization/
5.	intermethod comparison/
6.	placebo.ti,ab.
7.	(compare or compared or comparison).ti.
8.	((evaluated or evaluate or evaluating or assessed or assess) and (compare or compared or comparing or comparison)).ab.
9.	(open adj label).ti,ab.
10.	((double or single or doubly or singly) adj (blind or blinded or blindly)).ti,ab.
11.	double blind procedure/
12.	parallel group1.ti,ab.
13.	(crossover or cross over).ti,ab.
14.	((assign or match or matched or allocation) adj5 (alternate or group1 or intervention1 or patient1 or subject1 or participant 1)).ti,ab.
15.	(assigned or allocated).ti,ab.
16.	(controlled adj7 (study or design or trial)).ti,ab.
17.	(volunteer or volunteers).ti,ab.
18.	human experiment/
19.	trial.ti.
20.	or/1–19
21.	(random adj sampl adj7 (“cross section ” or questionnaire 1 or survey or database 1)).ti,ab. not (comparative study/or controlled study/or randomized controlled.ti,ab. or randomly assigned.ti,ab.)
22.	Cross-sectional study/not (exp randomized controlled trial/or controlled clinical study/or controlled study/or randomized controlled.ti,ab. or control group 1.ti,ab.)
23.	(((case adj control) and random) not randomized controlled).ti,ab.
24.	Systematic review.ti,ab. not (trial or study).ti.
25.	(nonrandom not random).ti,ab.
26.	“random field”.ti,ab.
27.	(random cluster adj3 sampl).ti,ab.
28.	(review.ab. and review.pt.) not trial.ti.
29.	“we searched”.ab. and (review.ti. or review.pt.)
30.	“update review”.ab.
31.	(databases adj4 searched).ab.
32.	(rat or rats or mouse or mice or swine or porcine or murine or sheep or lambs or pigs or piglets or rabbit or rabbits or cat or cats or dog or dogs or cattle or bovine or monkey or monkeys or trout or marmoset1).ti. and animal experiment/
33.	Animal experiment/not (human experiment/or human/)
34.	or/21–33
35.	20 not 34
36.	colchicine derivative/or colchicine/or colchicine.mp.
37.	acute myocardial infarction.mp. or acute heart infarction/
38.	unstable angina pectoris/or acute coronary syndrome.mp. or heart infarction/or coronary artery thrombosis/or acute coronary syndrome/or acute heart infarction/
39.	myocardial infarction.mp. or heart infarction/
40.	37 or 38 or 39
41.	35 and 36 and 40
**Medline search**	
1.	exp randomized controlled trial/
2.	controlled clinical trial.pt.
3.	randomized.ab.
4.	placebo.ab.
5.	drug therapy.fs.
6.	randomly.ab.
7.	trial.ab.
8.	groups.ab.
9.	or/1–8
10.	colchicine.mp. [mp = title, abstract, heading word, drug trade name, original title, device manufacturer, drug manufacturer, device trade name, keyword heading word, floating subheading word, candidate term word]
11.	acute myocardial infarction.mp. [mp = title, abstract, heading word, drug trade name, original title, device manufacturer, drug manufacturer, device trade name, keyword heading word, floating subheading word, candidate term word]
12.	heart attack.mp. [mp = title, abstract, heading word, drug trade name, original title, device manufacturer, drug manufacturer, device trade name, keyword heading word, floating subheading word, candidate term word]
13.	unstable angina pectoris/or acute coronary syndrome.mp. or heart infarction/or coronary artery thrombosis/or acute coronary syndrome/or acute heart infarction/
**Cochrane search**	
Search Name:	“myocardial infarction” OR “acute coronary syndrome” OR “unstable angina” OR “heart attack” in Title Abstract Keyword AND colchicine in Title Abstract Keyword (Word variations have been searched)
**Clinical trials.gov search**	
Search Name:	Acute Myocardial Infarction OR acute coronary syndrome OR unstable angina OR Heart Attack|Colchicine

**Table A3:** PRISMA checklist [13].

Section and Topic	Item #	Checklist Item	Location Where Item is Reported
**TITLE**			
Title	1	Identify the report as a systematic review.	p. 1
**ABSTRACT**			
Abstract	2	See the PRISMA 2020 for Abstracts checklist.	pp. 1–2
**INTRODUCTION**			
Rationale	3	Describe the rationale for the review in the context of existing knowledge.	pp. 2–3
Objectives	4	Provide an explicit statement of the objective(s) or question(s) the review addresses.	p. 2
**METHODS**			
Eligibility criteria	5	Specify the inclusion and exclusion criteria for the review and how studies were grouped for the syntheses.	p. 3; Table A1 (p. 13)
Information sources	6	Specify all databases, registers, websites, organisations, reference lists and other sources searched or consulted to identify studies. Specify the date when each source was last searched or consulted.	p. 3; Table A1 (p. 13)
Search strategy	7	Present the full search strategies for all databases, registers and websites, including any filters and limits used.	p. 3; Table A2 (pp. 14–15)
Selection process	8	Specify the methods used to decide whether a study met the inclusion criteria of the review, including how many reviewers screened each record and each report retrieved, whether they worked independently, and if applicable, details of automation tools used in the process.	p. 3; Table A3 (p. 16)
Data collection process	9	Specify the methods used to collect data from reports, including how many reviewers collected data from each report, whether they worked independently, any processes for obtaining or confirming data from study investigators, and if applicable, details of automation tools used in the process.	p. 3
Data items	10a	List and define all outcomes for which data were sought. Specify whether all results that were compatible with each outcome domain in each study were sought (e.g., for all measures, time points, analyses), and if not, the methods used to decide which results to collect.	p. 3; Table 1 (pp. 4–5)
	10b	List and define all other variables for which data were sought (e.g., participant and intervention characteristics, funding sources). Describe any assumptions made about any missing or unclear information.	p. 3; Table 1 (pp. 4–5)
Study risk of bias assessment	11	Specify the methods used to assess risk of bias in the included studies, including details of the tool(s) used, how many reviewers assessed each study and whether they worked independently, and if applicable, details of automation tools used in the process.	p. 3; Table 2 (p. 5); Table A5 and Table A6 (p. 18)
Effect measures	12	Specify for each outcome the effect measure(s) (e.g., risk ratio, mean difference) used in the synthesis or presentation of results.	pp. 3–4; Table 3 (pp. 5–6); Table A10 (p. 21)
Synthesis methods	13a	Describe the processes used to decide which studies were eligible for each synthesis (e.g., tabulating the study intervention characteristics and comparing against the planned groups for each synthesis (item #5)).	pp. 3–4; Table A1 (p. 13)
	13b	Describe any methods required to prepare the data for presentation or synthesis, such as handling of missing summary statistics, or data conversions.	No such methods were needed
	13c	Describe any methods used to tabulate or visually display results of individual studies and syntheses.	pp. 3–4; Figure 1, Figure 2, Figure 3, Figure 4, Figure 5, Figure 6 and Figure 7 (pp. 6–8); Table 1 (pp. 4–5); Table 2 and Table 3 (pp. 5–7)
	13d	Describe any methods used to synthesize results and provide a rationale for the choice(s). If meta-analysis was performed, describe the model(s), method(s) to identify the presence and extent of statistical heterogeneity, and software package(s) used.	pp. 3–4; Table A10 (p. 21)
	13e	Describe any methods used to explore possible causes of heterogeneity among study results (e.g., subgroup analysis, meta-regression).	p. 4; p. 8; Table A11 (p. 22)
	13f	Describe any sensitivity analyses conducted to assess robustness of the synthesized results.	p. 4; p. 8; Table A12 (pp. 22–23)
Reporting bias assessment	14	Describe any methods used to assess risk of bias due to missing results in a synthesis (arising from reporting biases).	p. 4; pp. 6–7 (Figure 1)
Certainty assessment	15	Describe any methods used to assess certainty (or confidence) in the body of evidence for an outcome.	p. 4; pp. 5–6 (Table 3); Table A7, Table A8 and Table A9 (pp. 19–20)
**RESULTS**			
Study selection	16a	Describe the results of the search and selection process, from the number of records identified in the search to the number of studies included in the review, ideally using a flow diagram.	p. 4; Table A3 (p. 16)
	16b	Cite studies that might appear to meet the inclusion criteria, but which were excluded, and explain why they were excluded.	p. 4; Table A4 (p. 17)
Study characteristics	17	Cite each included study and present its characteristics.	pp. 4–5 (Table 1)
Risk of bias in studies	18	Present assessments of risk of bias for each included study.	p. 5 (Table 2); Table A5 and Table A6 (p. 18)
Results of individual studies	19	For all outcomes, present, for each study: (a) summary statistics for each group (where appropriate) and (b) an effect estimate and its precision (e.g., confidence/credible interval), ideally using structured tables or plots.	pp. 4–8; Figure 2, Figure 3, Figure 4, Figure 5, Figure 6 and Figure 7 (pp. 7–8); Table A7 and Table A8 (pp. 21–23)
Results of syntheses	20a	For each synthesis, briefly summarise the characteristics and risk of bias among contributing studies.	pp. 5–7 (Table 2 and Table 3)
	20b	Present results of all statistical syntheses conducted. If meta-analysis was done, present for each the summary estimate and its precision (e.g., confidence/credible interval) and measures of statistical heterogeneity. If comparing groups, describe the direction of the effect.	pp. 7–8; Figure 2, Figure 3, Figure 4, Figure 5, Figure 6 and Figure 7 (pp. 7–8); Table A10 (p. 21)
	20c	Present results of all investigations of possible causes of heterogeneity among study results.	p. 8 (diabetes subgroup); Table A11 (p. 22)
	20d	Present results of all sensitivity analyses conducted to assess the robustness of the synthesized results.	p. 8; Table A12 (pp. 22–23)
Reporting biases	21	Present assessments of risk of bias due to missing results (arising from reporting biases) for each synthesis assessed.	pp. 6–7 (Figure 1)
Certainty of evidence	22	Present assessments of certainty (or confidence) in the body of evidence for each outcome assessed.	pp. 5–6 (Table 3); Table A7, Table A8 and Table A9 (pp. 19–20)
**DISCUSSION**			
Discussion	23a	Provide a general interpretation of the results in the context of other evidence.	pp. 8–10
	23b	Discuss any limitations of the evidence included in the review.	pp. 9–10
	23c	Discuss any limitations of the review processes used.	p. 10
	23d	Discuss implications of the results for practice, policy, and future research.	pp. 10–11
**OTHER INFORMATION**			
Registration and protocol	24a	Provide registration information for the review, including register name and registration number, or state that the review was not registered.	pp. 2–3, 11
	24b	Indicate where the review protocol can be accessed, or state that a protocol was not prepared.	pp. 2–3 (PROSPERO registration/URL in abstract); p. 11
	24c	Describe and explain any amendments to information provided at registration or in the protocol.	No substantive amendments were made after PROSPERO registration
Support	25	Describe sources of financial or non-financial support for the review, and the role of the funders or sponsors in the review.	pp. 10–11
Competing interests	26	Declare any competing interests of review authors.	p. 11
Availability of data, code and other materials	27	Report which of the following are publicly available and where they can be found: template data collection forms; data extracted from included studies; data used for all analyses; analytic code; any other materials used in the review.	p. 11

From Ref. [13]. For more information, visit http://www.prisma-statement.org/ (accessed on 15 September 2025).

**Table A4:** Table of relevant studies on the effect of colchicine in acute myocardial infarction—list of excluded studies with reasons (full-text stage).

#	Author	Acronym	Patients	Colchicine Regimen	Type of ACS	Primary Outcome	Results
1	Akrami et al. 2021 [25]	NA	249 (120/129)	0.5 mg starting the first day after ACS, for 6 months	UA, STEMI, NSTEMI	MACE	Lower rates of MACEs in colchicine group vs placebo (8 [6.7%] vs. 28 [21.7%]).
2	Bouleti et al. 2024 [26]	COVERT-MI	192	Follow-up analysis of the prespecified secondary clinical endpoints at 1 year	STEMI	MACE	No difference at 1 year
	Mewton et al. 2021 [27]	COVERT-MI	192	2 mg loading, 0.5 ×2 for 5 days	STEMI	Infarct size; MACE- secondary	MACE was not the primary outcome
3	Hosseini et al. 2022 [28]	PODCAST PCI	451 (229/222)	1 mg loading, 0.5 mg until discharge	STEMI	No reflow. MACE- secondary	MACE was not the primary outcome
4	Huet et al. 2024 [29]	COLD MI	54	1 mg per day for 30 days, initiated within 48 h after revascularization	STEMI	Percentage of myocardial denervation measured by SPECT at 6-m follow-up. MACE- secondary, not reported	MACE was not the primary outcome
5	Shah et al. 2020 [30] Shah et al. 2023 [31]	Colchicine-PCI	400 206/194 (50% ACS)	1.8 mg—Preprocedural	ACS in 50% of PCI patients	PCI-related myocardial injury, MACE- secondary	Key secondary outcomes of 30-day MACE and PCI-related MI did not differ. Same at 3.3 years
6	Khiali et al. 2024 [32]	NA	106 36/35/35	Empagliflozin 10 mg/day, empagliflozin 10 mg/day + colchicine 0.5 mg ×2 daily, or empagliflozin 25 mg/day within 72 h after primary PCI	Recent STEMI (72 h)	Change in NYHA class, CRP MACE -tertiary/safety outcome	No change
7	Talasaz et al. 2019 [33]	NA	196 (95/101)	Colchicine before PCI	STEMI	(TIMI) score; TIMI myocardial perfusion grade (TMPG), and TIMI frame count (TFC). MACE- secondary	MACE was not the primary outcome

## Appendix B. Risk of Bias Assessment

**Table A5:** Colchicine trials on acute coronary syndromes: risk of bias. Summary of RoB (Traffic Bar). COPS, Colchicine in Patients With Acute Coronary Syndrome; COLCOT, Colchicine Cardiovascular Outcomes Trial; CLEAR SYNERGY (OASIS-9), Colchicine in Acute Myocardial Infarction (OASIS-9 trial).

Trial	Low Risk	Some Concerns	High Risk
COLCOT	█████████████████████████████ (100%)		
CLEAR SYNERGY	█████████████████████████████ (100%)		
COPS	████████████░░░░░░░░░░░░░░░ (60%)		

**Table A6:** Risk of bias assessment (Cochrane RoB 2.0) by domain and overall.

Trial/Domain	Randomization	Deviations from Interventions	Missing Outcome Data	Measurement of Outcomes	Selection of Reported Result	Overall RoB
COPS	Low	Low	Some concerns	Low	Some concerns	Some concerns
COLCOT	Low	Low	Low	Low	Low	Low
CLEAR SYNERGY	Low	Low	Low	Low	Low	Low

Abbreviations: COPS, Colchicine in Patients with Acute Coronary Syndrome; COLCOT, Colchicine Cardiovascular Outcomes Trial; CLEAR SYNERGY (OASIS-9), Colchicine in Acute Myocardial Infarction (OASIS-9 trial).

## Appendix C. Colchicine Trials on Acute Coronary Syndromes GRADE Evidence Profiles

**Table A7:** GRADE evidence profile of COLCOT, Colchicine Cardiovascular Outcomes Trial (Post-MI, n = 4745).

Outcome	Placebo	Colchicine	Relative Effect	Participants	Certainty (GRADE)	Comments
MACEs (CV death, MI, stroke, urgent revascularization)	9.6%	6.8%	HR 0.77 (0.61–0.96)	4745 (1 RCT)	High	Significant reduction; low RoB.
Non-fatal MI	4.0%	2.7%	HR 0.66 (0.50–0.87)	4745	High	Clear benefit.
CV death	1.0%	1.0%	HR 0.99 (0.57–1.72)	4745	High	No difference.
Safety—GI events	7.0%	9.7%	*p* < 0.001	4745	High	Increased GI adverse events.

**Table A8:** GRADE evidence profile of CLEAR SYNERGY, Colchicine in Acute Myocardial Infarction (OASIS 9, n = 7062).

Outcome	Placebo	Colchicine	Relative effect	Participants	Certainty (GRADE)	Comments
MACE	9.3%	9.1%	HR 0.99 (0.85–1.16)	7062	High	Neutral effect.
Non-fatal MI	3.1%	2.9%	HR 0.88 (0.66–1.17)	7062	High	CI crosses unity.
CV death	3.2%	3.3%	HR 1.03 (0.80–1.34)	7062	High	No difference.
Safety—Diarrhea	6.6%	10.2%	*p* < 0.001	7062	High	GI adverse events increased.

**Table A9:** GRADE evidence profile of COPS, Colchicine in Patients With Acute Coronary Syndrome (ACS, n = 795).

Outcome	Placebo	Colchicine	Relative Effect /Comment	Participants	Certainty (GRADE)	Comments
Composite MACE	13.5%	8.1%	Approximate OR 0.56 (95% CI 0.35–0.89); log-rank *p* = 0.02	795	Low-Moderate	Significant reduction at 24 months; interpret cautiously given trial size
All-cause mortality	1.0%	2.3%	HR 2.28 (95% CI 0.7–7.4); *p* = 0.17	795	Low	Numerical imbalance, not statistically significant at 24 months
Non-CV death	0.5%	1.3%	*p* = 0.024	795	Low	Potential harm.
Urgent revasc.	1.5%	0.37%	*p* = 0.037	795	Low-Moderate	Interpret with caution.

**Table A10:** Pooled meta-analysis summary by outcome (primary, secondary and subgroup outcomes).

Outcome	k	Events (e)	Model	Pooled OR (95% CI)	z	*p*	I^2^ (%)	τ^2^	Heterogeneity (Q, df, *p*)
MACE (pr. outcome)	3	1036	Common	0.8732 [0.7688; 0.9917]	−2.09	0.0368	71.3	0.0438	Q = 6.96, df = 2, *p* = 0.0308
			Random	0.7955 [0.5985; 1.0573]	−1.58	0.1150	71.3	0.0438	(same as above)
CV death (sec. outcome)	3	280	Common	1.0173 [0.8022; 1.2902]	0.14	0.8873	0.0	0.0000	Q = 1.09, df = 2, *p* = 0.5797
			Random	1.0173 [0.8022; 1.2902]	0.14	0.8873	0.0	0.0000	(same as above)
Myocardial infarction (sec. outcome)	3	444	Common	0.8809 [0.7288; 1.0646]	−1.31	0.1895	0.0	<0.0001	Q = 1.38, df = 2, *p* = 0.5017
			Random	0.8809 [0.7288; 1.0646]	−1.31	0.1895	0.0	<0.0001	(same as above)
Revasc. (sec. outcome)	3	424	Common	0.8238 [0.6783; 1.0005]	−1.96	0.0506	85.2	0.3521	Q = 13.52, df = 2, *p* = 0.0012
			Random	0.5521 [0.2627; 1.1604]	−1.57	0.1170	85.2	0.3521	(same as above)
Stroke (sec.outcome)	3	127	Common	0.8417 [0.5933; 1.1942]	−0.97	0.3343	78.6	0.4407	Q = 9.33, df = 2, *p* = 0.0094
			Random	0.5940 [0.2449; 1.4408]	−1.15	0.2493	78.6	0.4407	(same as above)
MACE (diabetes subgroup)	3	272	Common	0.8055 [0.6258; 1.0369]	−1.68	0.0933	0.0	0.0000	Q = 1.07, df = 2, *p* = 0.5851
			Random	0.8055 [0.6258; 1.0369]	−1.68	0.0933	0.0	0.0000	(same as above)

## Appendix D. Sensitivity and Subgroup Analyses

**Table A11:** Diabetes subgroup—individual trial effects.

Trial	Measure	Colchicine Group Events	Placebo Group Events	Effect	95% CI
COPS	OR	5/75	7/76	0.71	0.22–2.29
COLCOT	OR	38/462	58/497	0.68	0.45–1.04
CLEAR	OR	79/658	85/645	0.90	0.65–1.25

Abbreviations: COPS, Colchicine in Patients With Acute Coronary Syndrome; COLCOT, Colchicine Cardiovascular Outcomes Trial; CLEAR SYNERGY (OASIS-9), Colchicine in Acute Myocardial Infarction (OASIS-9 trial).

**Table A12:** Leave-one-out sensitivity analysis.

Outcome	Removed Trial	k (rem)	Common OR (95% CI)	Random OR (95% CI)	I^2^ (%)	τ^2^	Significance Change (CE)	Significance Change (RE)
**MACE (primary)**	COPS et al.	2	0.9068 [0.7941; 1.0355]	0.8794 [0.6856; 1.1280]	67.9	0.0222	Yes	No
**MACE (primary)**	COLCOT et al.	2	0.9246 [0.7943; 1.0762]	0.7799 [0.4580; 1.3281]	80.5	0.1215	Yes	No
**MACE (primary)**	CLEAR et al.	2	0.7163 [0.5824; 0.8810]	0.7025 [0.5424; 0.9099]	23.2	0.01	No	Yes
**CV DEATH**	CLEAR et al.	2	0.91 [0.52; 1.60]	0.91 [0.52; 1.60]	0.0	0.0		
**CV DEATH**	COLCOT et al.	2	1.05 [0.81; 1.36]	1.05 [0.81; 1.36]	0.0	0.0		
**CV DEATH**	COPS et al.	2	1.00 [0.78; 1.27]	1.00 [0.78; 1.27]	0.0	0.0		
**MI**	CLEAR et al.	2	0.85 [0.65; 1.11]	0.85 [0.65; 1.11]	0.0	0.0		
**MI**	COLCOT et al.	2	0.87 [0.68; 1.11]	0.86 [0.65; 1.13]	7.1	0.0046		
**MI**	COPS et al.	2	0.91 [0.75; 1.12]	0.91 [0.75; 1.12]	0.0	0.0		
**REVASC**	CLEAR et al.	2	0.44 [0.28; 0.69]	0.37 [0.15; 0.89]	49.9	0.2301		
**REVASC**	COLCOT et al.	2	0.94 [0.75; 1.17]	0.49 [0.10; 2.39]	84.5	1.1296		
**REVASC**	COPS et al.	2	0.88 [0.72; 1.07]	0.73 [0.38; 1.41]	83.8	0.1908		
**STROKE**	CLEAR et al.	2	0.31 [0.14; 0.69]	0.31 [0.14; 0.69]	0.0	0.0		
**STROKE**	COLCOT et al.	2	1.07 [0.72; 1.58]	0.88 [0.37; 2.10]	46.6	0.2293		
**STROKE**	COPS et al.	2	0.93 [0.64; 1.36]	0.59 [0.14; 2.52]	86.5	0.949		

Abbreviations: COPS, Colchicine in Patients With Acute Coronary Syndrome; COLCOT, Colchicine Cardiovascular Outcomes Trial; CLEAR SYNERGY (OASIS-9), Colchicine in Acute Myocardial Infarction (OASIS-9 trial).

**Table A13:** Exploratory analysis stratified by longest available follow-up for the primary outcome.

Follow-Up Category	Trial	Total Participants, n	Trial-Level Primary Outcome Summary	Interpretation
~24 months	COPS (prespecified two-year follow-up)	795	OR 0.56 (95% CI 0.35–0.89);	Significant reduction
~24 months	COLCOT	4745	OR 0.77 (95% CI 0.61–0.96)	Significant reduction
~24 months	Exploratory pooled estimate (COPS 24 months + COLCOT)	5540	Approximate common-effect OR 0.71	Consistent benefit
36 months	CLEAR SYNERGY (OASIS-9)	7062	OR 0.99 (95% CI 0.85–1.16)	Neutral effect

Abbreviations: COPS, Colchicine in Patients With Acute Coronary Syndrome; COLCOT, Colchicine Cardiovascular Outcomes Trial; CLEAR SYNERGY (OASIS-9), Colchicine in Acute Myocardial Infarction (OASIS-9 trial).
